# Integrating trials into a whole-population cohort of children and parents: statement of intent (trials) for the Generation Victoria (GenV) cohort

**DOI:** 10.1186/s12874-020-01111-x

**Published:** 2020-09-24

**Authors:** Melissa Wake, Yanhong Jessika Hu, Hayley Warren, Margie Danchin, Michael Fahey, Francesca Orsini, Maurizio Pacilli, Kirsten P. Perrett, Richard Saffery, Andrew Davidson

**Affiliations:** 1 Murdoch Children’s Research Institute, The Royal Children’s Hospital, 50 Flemington Road, Parkville, VIC 3052 Australia; 2 Department of Paediatrics, The University of Melbourne, Parkville, VIC 3052 Australia; 3 The Royal Children’s Hospital, Parkville, VIC 3052 Australia; 4 Department of Paediatrics, Monash University, Clayton, VIC 3168 Australia; 5 Monash Children’s Hospital, Clayton, VIC 3168 Australia

**Keywords:** Research methodology, Randomization, Registry trials, Multiple baseline randomized trials, Trials within cohorts, Population studies, Generation Victoria (GenV), Clinical trial as topic, Children, Intervention

## Abstract

**Background:**

Very large cohorts that span an entire population raise new prospects for the conduct of multiple trials that speed up advances in prevention or treatment while reducing participant, financial and regulatory burden. However, a review of literature reveals no blueprint to guide this systematically in practice. This Statement of Intent proposes how diverse trials may be integrated within or alongside Generation Victoria (GenV), a whole-of-state Australian birth cohort in planning, and delineates potential processes and opportunities.

**Methods:**

Parents of all newborns (estimated 160,000) in the state of Victoria, Australia, will be approached for two full years from 2021. The cohort design comprises four elements: (1) consent soon after birth to follow the child and parent/s until study end or withdrawal; retrospective and prospective (2) linkage to clinical and administrative datasets and (3) banking of universal and clinical biosamples; and (4) GenV-collected biosamples and data. GenV-collected data will focus on overarching outcome and phenotypic measures using low-burden, universal-capable electronic interfaces, with funding-dependent face-to-face assessments tailored to universal settings during the early childhood, school and/or adult years.

**Results:**

For population or registry-type trials within GenV, GenV will provide all outcomes data and consent via traditional, waiver, or Trials Within Cohorts models. Trials alongside GenV consent their own participants born within the GenV window; GenV may help identify potential participants via opt-in or opt-out expression of interest. Data sharing enriches trials with outcomes, prior data, and/or access to linked data contingent on custodian’s agreements, and supports modeling of causal effects to the population and between-trials comparisons of costs, benefits and utility. Data access will operate under the Findability, Accessibility, Interoperability, and Reusability (FAIR) and Care and Five Safes Principles. We consider governance, ethical and shared trial oversight, and expectations that trials will adhere to the best practice of the day.

**Conclusions:**

Children and younger adults can access fewer trials than older adults. Integrating trials into mega-cohorts should improve health and well-being by generating faster, larger-scale evidence on a longer and/or broader horizon than previously possible. GenV will explore the limits and details of this approach over the coming years.

## Background

Randomized controlled trials (RCT) provide high-quality evidence with regards to the effectiveness of therapies and prevention and are critical to guide translation and optimal resource allocation. The traditional parallel-arms trial design is a stand-alone initiative for which each trial identifies a specific question and sample, obtains funding, consents and randomizes subjects to two or more different treatments or interventions, follows the groups in parallel and collects the outcome data. There are many variations – for example, cluster vs. individual randomization, and stepped-wedge, adaptive and cross-over designed.

Stand-alone randomized trials are challenging, slow and costly [1]. More than two-thirds of multi-center, publicly funded UK trials do not recruit their target number of patients within the specified timeframe [2]. Consequently, trials are often underpowered, require an extension with additional costs, and encounter delayed translation into clinical or preventive practice, or are never completed. Furthermore, most trials interrogate a small number of hypotheses in restricted groups over a short time frame (when a long-term benefit is often the real goal), often with considerable heterogeneity between trials in samples, methods and outcomes. Collectively, this results in financial and scientific inefficiencies and a lack of generalizability and translatability [3]. This situation is particularly problematic for children [4], whose evidence base (and therefore care) lags due to a paucity of trials [5, 6].

One efficient and generalizable solution is to embed trials in existing data collection structures such as registries, electronic health records, and administrative databases [7, 8]. There are many examples of this approach. High-quality registries focus on full, unbiased condition ascertainment with standardized outcomes embedded into clinical care. These can support registry trials, whereby a registry participant meets a trial’s eligibility criteria, is consented, randomized and accrues trial outcomes that are usually fully embedded in the registry. Point-of-care trials embed trial processes (like randomization, ascertainment of outcomes) into the clinical care process, increasingly by effectively using the electronic medical record (EMR). Large, simple trials may compare interventions already in standard care, but for which evidence of superiority or equivalence is not available [9]. As individual risk is low, these may include opt-out (with inclusion the default) or waiver of consent, point-of-care randomization (see above), and/or short information statements. Registry, point-of-care and large, simple trial elements may co-occur in a single trial.

A recent development to leverage even greater health gain from single registries is to coordinate simultaneous registry trials using pre-specified master protocols. These may test the impact of targeted therapy on multiple diseases (basket trials), of multiple therapies on a single disease (umbrella trials), or of several interventions against a common control (platform trials, also known as multi-arm multi-stage (MAMS) trials) [10]. Park’s 2019 ‘landscape’ analysis reported rapid growth in master protocols over the last 5 years. However, very few of these trials target children, and most are in highly specialized fields. Thus, of the 83 master protocols identified (49 basket, 18 umbrella, 16 platform; 44 in the US), most were in adults (69/83, 83%), exploratory, and designed to examine experimental drugs (82/83, 99%) in the field of oncology (76/83, 92%) [11]. Challenges include stakeholder coordination, infrastructure and governance requirements, and the integration and complexities of the pre-specified trial and analysis design [10].

Multiple trials can also be conducted within longitudinal epidemiological cohorts. The traditional role of a cohort study is to observe incidence, prevalence, trajectories, natural history and exposure-outcome associations. However, their sampling design and longer outcome horizons may also be appealing to trials, for which the cohorts can act essentially as population-based registries. One advantage is that the trial sample can be compared to a broader population in terms of baseline characteristics and natural history of a condition of interest and its short and long term outcomes. Trials may be conducted in parallel with the cohort and across a wide range of patient and participant groups, as in Western Australia’s ORIGINS Project [12]; with appropriate consent, a cohort may subsequently provide pre-randomization (e.g., genetic) and some or all outcomes data for trials. A second model has been variously labelled Zelen trials [13], cohort multiple randomized controlled trials (cmRCTs) [3, 14], and Trials Within Cohorts (TWiCS), as in England’s Born in Bradford Better Start cohort [7]. In TWiCS, cohort participants consent to contribute control data to future unspecified trials at recruitment into the cohort itself, with only participants randomly allocated to the intervention arm then asked for informed consent into any given TWiCs trial. Despite the potential for allocation bias, they can be efficient and achieve valid [14] and meaningful outcomes [15, 16], and can evaluate the impact of ‘stacked’ interventions [7] approximating how parents and children naturalistically navigate needed services. A third potential model is that of master protocols as above that predefine from the outset and coordinate a set of trials that may occur. We are aware of two potential examples for healthy cohorts: The developing global ALPHA Collaboration aims to embed large platform trials in routine care to improve maternal and perinatal health, while HeLTI (Healthy Life Trajectories Initiative) comprises harmonized interventional periconception cohorts in China, South Africa, India and Canada testing pre-planned, stacked interventions over a 10-year period aiming to prevent obesity and other non-communicable diseases in over 20,000 children.

If a cohort were to involve a sufficiently large and complete population, then it would be possible to enact a range of these ideas simultaneously, encompassing a wide range of unmet trial needs from rare diseases through to population and health services research. The forthcoming Generation Victoria (GenV) provides an opportunity to explore and operationalize these ideas. GenV is a state-wide cohort that will approach for recruitment, parents of all newborns (estimated 160,000) in the state of Victoria (population 6.5 million [17]), Australia, over two full years from 2021. Its goal is to generate translatable evidence (prediction, prevention, treatments, services and policy) to improve the future wellbeing of all children and adults and to reduce future disease burden. However, there is no existing road map in the international literature to achieve and encourage the flexible trials-GenV integration that could contribute to this goal. Here, we report on the preliminary processes and guidance we have developed so that trials can prospectively integrate within a substantial cohort (GenV) to maximize these opportunities. As a Statement of Intent, this paper differs from a master protocol in that it does not prespecify any one trial or trial design.

## Methods

### Administrative information

This Statement of Intent outlines the proposed principles and processes for the integration of future trials into GenV. Its development has been guided by the SPIRIT 2013 Statement [18] and the anticipated CONSORT extension for RCTs using cohorts and routinely collected health data.

The GenV cohort is currently in advanced design (see below). An AUD 24.5 million grant from the Paul Ramsay Foundation supports its infrastructure development, while a $16 million grant from the Victorian Government supports its design, cohort planning and implementation, stakeholder engagement and knowledge translation activities. Its sponsor-investigator is Professor Melissa Wake, who is also GenV’s Scientific Director. The Core Executive comprises Melissa Wake, Richard Saffery (Deputy Director, Biosciences), Sharon Goldfeld (Deputy Director, Equity and Knowledge Translation) and Kathryn North (Director, Murdoch Children’s Research Institute (MCRI)). The Directors regularly report to GenV’s Operational Advisory Committee and thence to the Board of the MCRI. Several advisory committees inform GenV, including a broad range of senior Victorian researchers comprising the Investigator Committee, and several Working Groups of which the Ethics & Governance and Trials Working Groups are relevant here. This relatively simple administrative structure may mature after successful cohort implementation. GenV has been endorsed by The Royal Children’s Hospital Human Research Ethics Committee (HREC 2019.011), including in-principle approval as a mechanism to support trials.

GenV proposes to work with trials that fulfil the administrative information requirements laid out in the SPIRIT 2013 Checklist (see Additional file 1) or equivalent at time of application. These cover title, trial registration, protocol with version control, funding sources and types, and roles and responsibilities of protocol contributors, sponsor/s and funder/s, and other individuals or groups overseeing the trial such as the coordinating center. These documents will contain the unique features of each trial’s design, participants, timelines and outcomes, as well as data sharing and other relationships with GenV.

### About GenV

At the time of writing, the GenV cohort is in advanced planning and, therefore, still evolving. A range of GenV summary documents are available for review on figshare (https://mcri.figshare.com/projects/Generation_Victoria/35822) [19]. Here, we outline GenV with only enough contextual detail to inform the context of the trials.

#### Purpose

GenV’s primary objective is to create very large, parallel whole-of-state birth and parent cohorts for discovery and interventional research. GenV data and biosamples can only be used for research that benefits human health.

#### Setting

GenV’s setting is the entire state of Victoria (population 6.5 million in 2019), Australia [17]. Because it may be relevant to trials that could be undertaken in a cohort of this magnitude, we provide some Victorian descriptive information here. In the 2016 Census, The median age of Victorian people was 37 years; 18.2% of its population were 0–14-year-old children, 15.6% were aged 65 years and over, and 49.1% were male [20]. Around 65% of Victorian residents were born in Australia. The most common ancestries were English (22.6%), Australian (21.1%), Irish (7.6%), Scottish (6.3%) and Chinese (4.7%), but its multi-ethnicity is reflected in more than 250 languages. Around 1% of the population identify as Aboriginal and/or Torres Strait Islander [20]. Like other Australians, Victorians are relatively affluent, with a median weekly pre-tax personal income for people aged 15 years and over of AUD 644 in 2016 [21]. However, a wide range of advantage-disadvantage exists, with 13% of Victoria’s population, and 18% of its children, living below the poverty line based on the 2016 Census data [22]. Over 60% of Australian parents report their child has at least one ongoing health or developmental problem at every age from age 2, rising to around 70% from age 8 to 15 years [23].

#### GenV design

GenV is a population-based cohort study that blends study-collected, study-enhanced and linked data. The cohort design comprises four elements: (1) consent soon after birth to follow the child and parent/s indefinitely until the study closes (no end date set at this point) or withdrawal, (2) retrospective and prospective linkage to clinical and administrative datasets, (3) banking of retrospective and prospective universal and clinical biosamples, and (4) GenV-collected biosamples and data.

#### GenV recruitment and consent

GenV proposes to recruit for two full years from 2021 in all of Victoria’s birthing hospitals (*n* = 70 at time of writing) [24], in which collectively around 83,000 babies are born each year. MCRI-employed recruiters aim to personally approach parents of all newborns for consent, with interpreting and translation support as needed. Children will have the opportunity to decide on their continued participation as they reach the age for legal consent. GenV’s consent includes parent permission for approved researchers to access GenV’s data, for data sharing between GenV and external trials, and for recontact to offer additional research opportunities.

#### Participants and inclusion/exclusion criteria

All children born in Victoria during the recruitment period whose parents/guardians have decisional capacity, and their parents, are eligible to take part. Participants who leave Victoria may continue to take part via linked and contributed data, and families may join GenV, who move into Victoria later and have children born within the recruitment period. However, in both of these instances, data may be incomplete.

## Results

### Principles for GenV and for trials within and alongside GenV

**Fig. 1:**
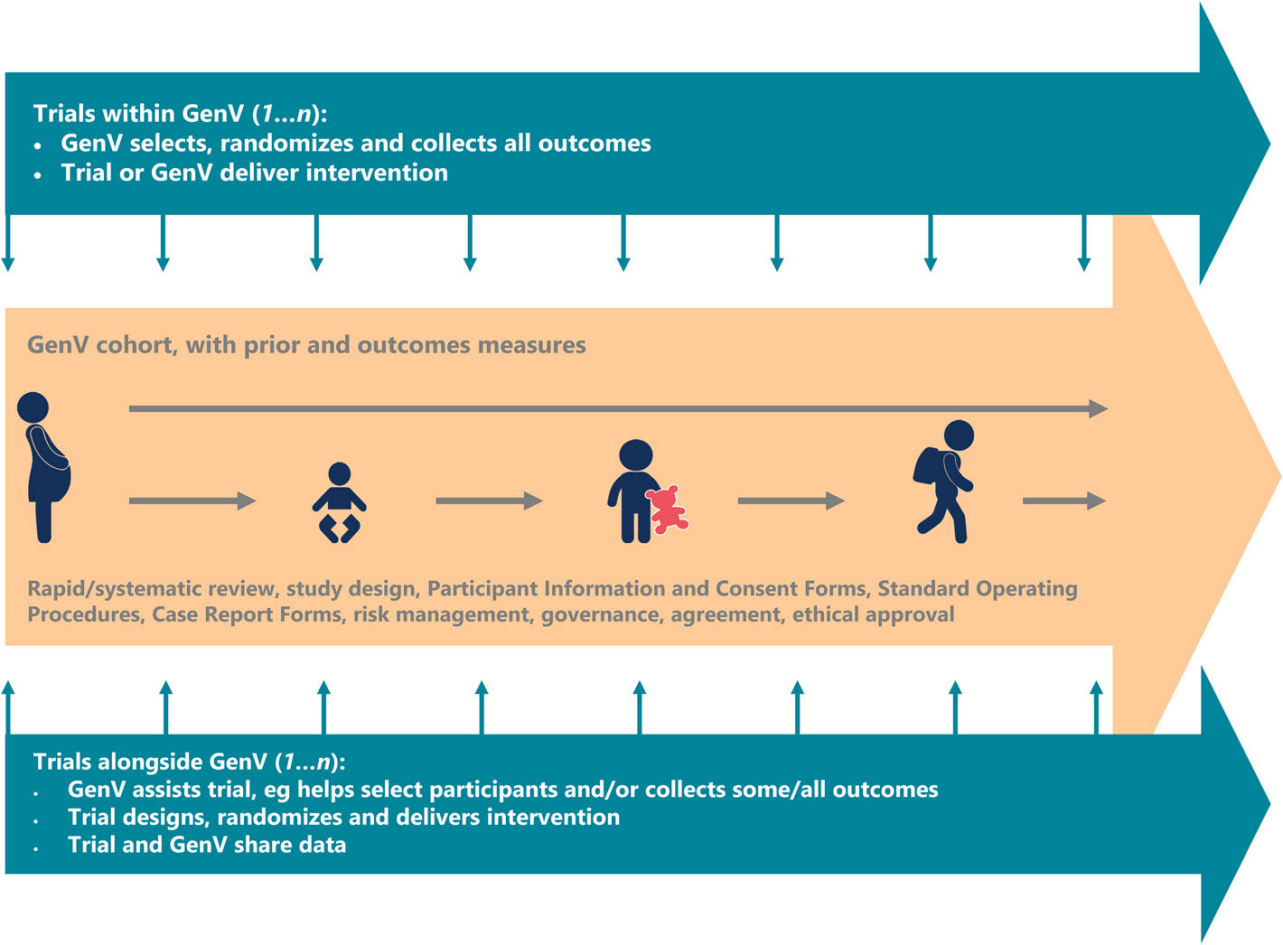
Relationship of the main cohort to trials within and alongside GenV

**Fig. 2:**
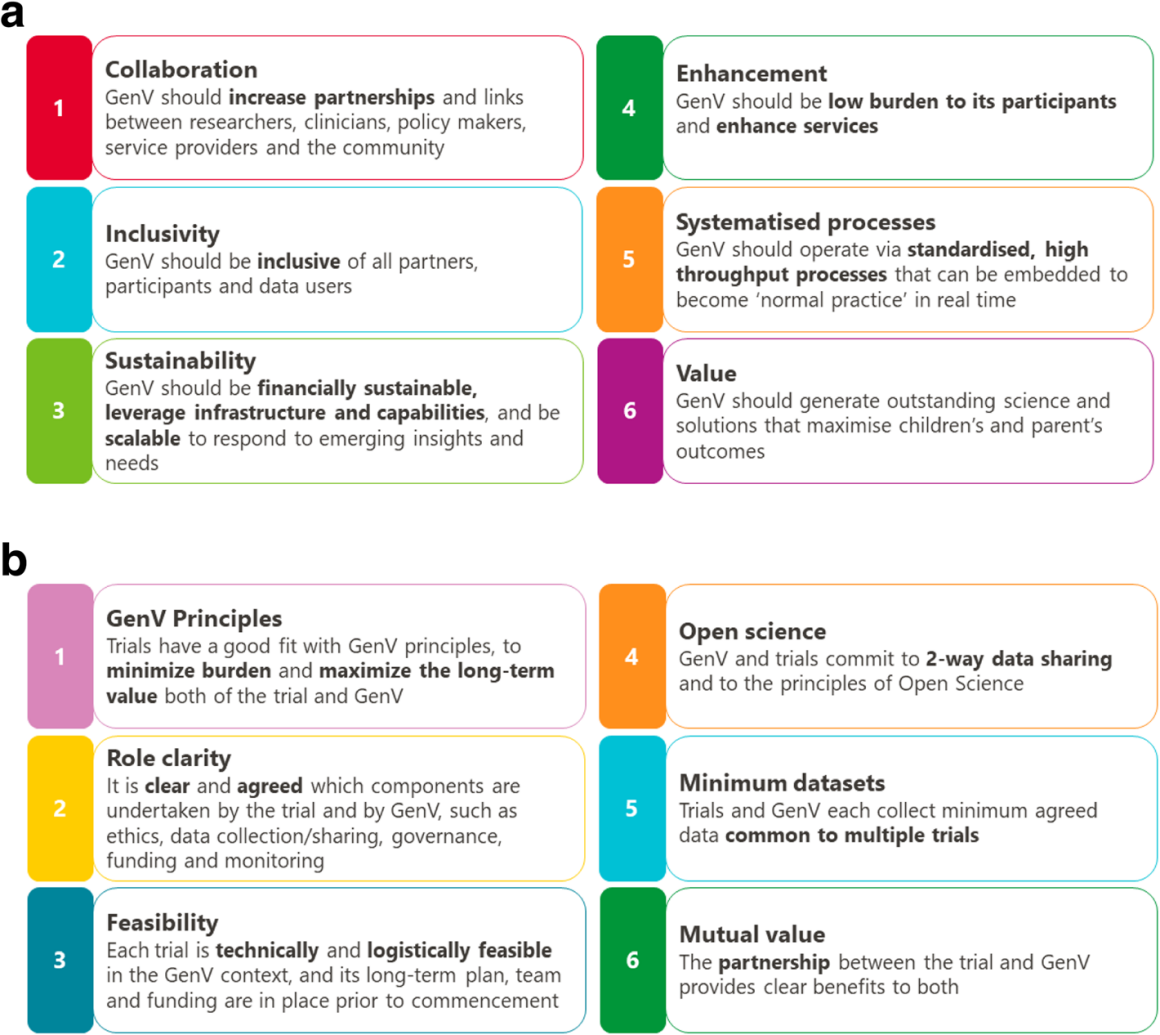
**a** GenV Principles. **b** Principles for Trials Within and Alongside GenV

Figure 1 is an infographic that shows the concept of how trials might integrate with GenV across the life course. All GenV activities (including those that relate to trials) are informed by the GenV principles, as outlined in Fig. 2(a): Collaboration, Inclusivity, Sustainability, Enhancement, Systemized Processes and Value. Therefore, it is implicit that all trials working prospectively within and alongside GenV would also be a good fit with these principles. We do not anticipate that this would impose an additional burden since funding bodies and international guidelines already stipulate best practice integral to trials, such as high-quality evidence of need (e.g. a PROSPERO-registered systematic or rapid review) and checklists (e.g. CONSORT, SPIRIT). All trials need their own ethical and other approvals before they can start. For maximal mutual benefit, we envisage that partnerships between GenV and trials will generally be prospective, i.e., worked out and agreed before the trial begins. We are currently developing processes to operationalize the trial-specific principles outlined in Fig. 2(b) in minimally burdensome ways. Other trials will continue as they have always done, independently and unrelated to GenV.

### Trial models and their relationships with GenV

**Fig. 3:**
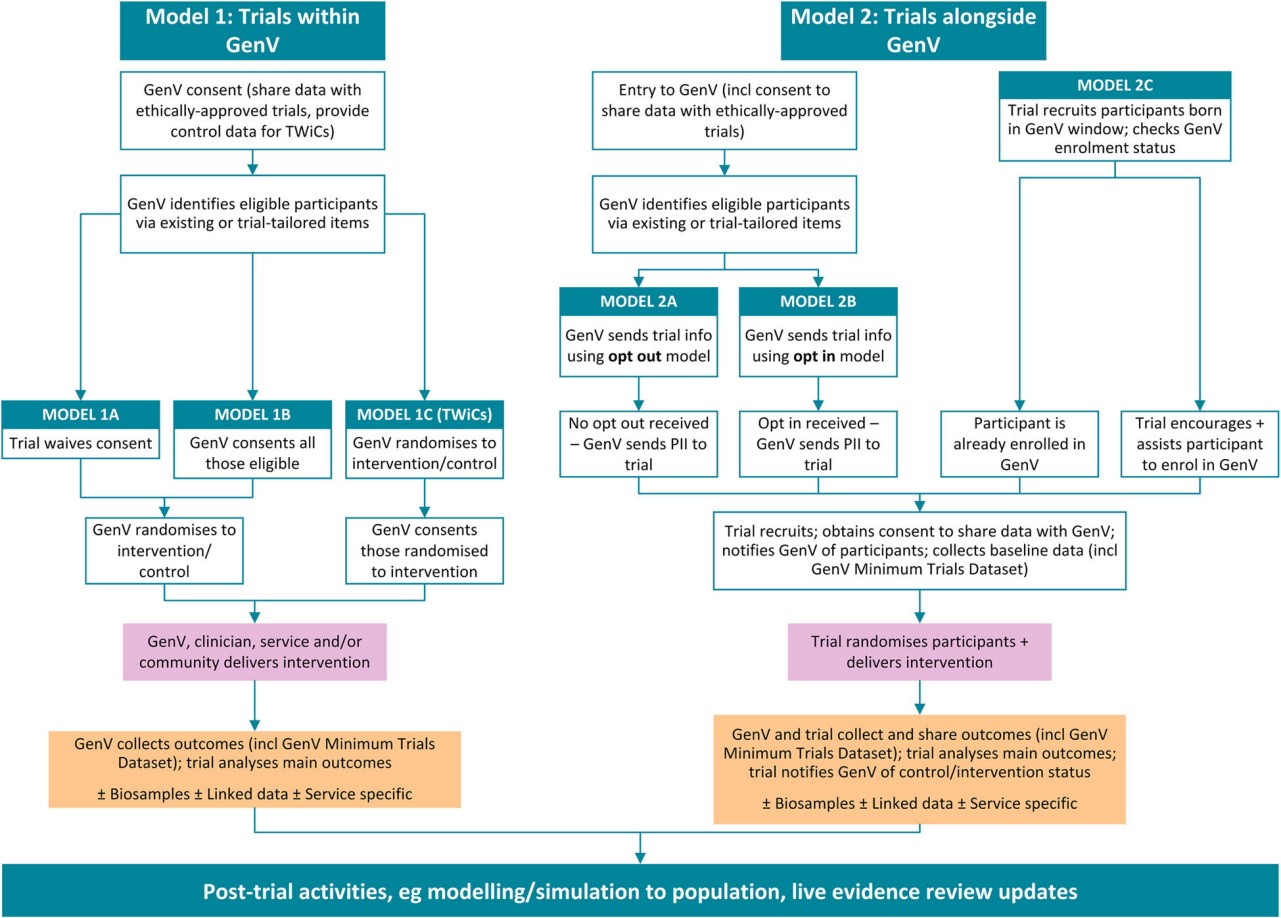
Statement of intent: process flowchart for trials within and alongside GenV

GenV may support trials within (Model 1) and trials alongside (Model 2) GenV, as outlined in Fig. 3; these models do not dictate the design of the trial (e.g., whether individually or cluster randomized).

Trials *within* GenV (Model 1) may be conducted as standard trials with opt-in, opt-out, or waiver of consent preceding randomization. Alternatively, TWiCs/cohort multiple RCTs may sometimes be considered whereby participants are randomized and then only those randomized to the intervention provide additional opt-in or opt-out consent.

Interventions for trials *alongside* GenV (Model 2) may be delivered either by GenV or by trials themselves, with the latter well suited to trials arising from EMRs and external registries for participants born in the GenV window. For Model 2, the trial collects the participant’s consent for two-way data sharing with GenV, including a minimum dataset of items common to multiple trials. Governance will be agreed upon before the trial implementation. For trials alongside GenV, all or some of the trial sample is also in GenV. In the latter situation, the GenV Principles and the data sharing and enhancing benefits of GenV would only apply to that subsample.

### Preparatory work

#### Content

We anticipate that most trials that integrate with GenV will be proposed by researchers outside GenV’s implementation team, who would, therefore, also define the content, ideally to have a good ‘fit’ with GenV. Some trialists are already actively approaching us with ideas for trials that would either be impossible without GenV or would benefit from otherwise inaccessible outcomes data. GenV hopes to elicit other possibilities via activities (to be developed) such as publicized annual open face-to-face and web-based fora to brainstorm and prioritize trial ideas, ideally involving a range of stakeholders including services, communities and families.

#### Working together

Good communication, transparency and agreement are vital and will underpin a Working Together Agreement between GenV and each trial (example shown in Additional file 2) developed following GenV’s rapid evidence review of large research-led partnerships [25]. Trialists and those responsible for GenV may at times have differing opinions on where the balance of benefit vs burden lies, and this will need to be considered openly. The benefit can be demonstrated by a rapid or systematic review supporting the need for the trial, ideally within the context of a ‘living’ review that can be readily updated over time, including with the results of the trial [26–28]. Burden relates not only to participants (consent, intervention content, follow-up and thence potential attrition) but also to impacts on GenV itself and its guiding principles.

GenV is not funded to conduct trials, which will require their own funding. GenV can currently provide limited support (including supporting trials to apply for funding) and is seeking dedicated funding to be able to provide additional support. In the meantime, activities such as determining eligible participants, consent, randomization and (potentially) intervention delivery as per Fig. 2, model 1 may need to be undertaken on a cost-recovery basis. For support in the form of expertise, GenV - rather than reinventing the wheel - proposes to connect proposed trials to local expert bodies such as the Melbourne Children’s Trials Centre, Monash University Trials Hub, NHMRC Clinical Trials Centre, Australian Clinical Trials Alliance (ACTA), and the Interdisciplinary Maternal Perinatal Australasian Collaborative Trials (IMPACT) network of maternal and perinatal trials.

#### Agreement to proceed with a trial-GenV collaboration

We anticipate an initial trial-GenV discussion that articulates the rationale of the intervention, trial design and research questions using the PICOT framework. If feasibility (potentially demonstrated through pilot studies) and mutual alignment appear likely [29], the trial would proceed to a partnering agreement that defines at least the following 8 items: 1) Which GenV trial model is being followed; 2) Design and high-level (or draft) protocol; 3) Timelines; 4) Data sharing and governance plans; 5) Status of ethical approval; 6) Communication with participants, including information statement and consent; 7) Trial oversight and 8) Capacity assessment, including trial quality, human resource and funding. We envisage that this discussion will be enabled by a yet-to-be-established GenV Trials Oversight Committee with cross-disciplinary expertise. Inclusion of consumers, including GenV participants, will be important to minimize participant burden and streamline data collection and trial conduct. The trial sponsor will usually be a representative of a university, research institute or similar organisation, but GenV does not preclude collaboration with commercial sponsors provided all its principles are met.

#### Maintenance

It is assumed that, throughout the trial, GenV will collect agreed data and maintain high retention while the trial will maintain independent quality, ethical and governance protocols in line with international standards. It is also assumed that GenV staff will collaborate with the trial management staff in order to understand, prevent and solve any day-to-day issues at the GenV end that may impact on the trial or GenV.

### Consent and randomization

#### Consent

Integration of trials with GenV to greatest effect will occur with appropriate consent wording in both the trial and GenV. At recruitment into GenV, parents provide consent for GenV to follow themselves and their child. As per the CONSORT Extension [30] for RCTs Using Cohorts and Routinely Collected Health Data, this includes consent to use their data for research purposes, with ongoing mechanisms to enable change in consent status (such as partial or full withdrawal or re-entry) any time after that. GenV’s full Parent Information & Consent Statement (PICF) is available for review [19]; its explicit trial-relevant wording is shown in Table 1 (a).

**Table 1:** PICF wording for (a) GenV to work with and (b) trials to work with GenV

(a) Wording in the GenV PICF that is specific to supporting trials:	
• “You may be offered the chance to take part in future ethically approved studies working with GenV …. You can always choose whether to take part.”	
• “GenV’s data can only be used for ethically approved research to improve health, development, or wellbeing for children and adults. Over time, researchers will use lots of different methods to answer new and important questions. Therefore, the value of your information will keep growing for many years.”	
• “Some GenV participants may join research trials testing new approaches. All trials need ethical approval. Who is offered the new approach is randomly picked, like tossing a coin. In some trials, only people offered the new approach are contacted about taking part. GenV data can be used to compare the outcomes of people who do and do not receive the new approach.”	
• “Trials … may ask your consent to share data with GenV, with ethics approval. We support this.”	
(b) Suggested wording for trials to include in their PICF, as appropriate to its degree of integration with GenV (Fig. 2):	
• “This trial is working with *[Model 2]*/part of *[Model 1]* the Generation Victoria (GenV) program. GenV is a research program open to all children living in Victoria and born over two years starting in 2021, and their parents. People in GenV can also be in trials testing new approaches to prevent, predict and treat important issues. This cuts down cost, effort and duplication. It also increases the value of trials. For example, by drawing on GenV data, a trial can look at more outcomes over a longer time than it could otherwise. You can read more about GenV here, and about the trials working with GenV here.”	
• *[Model 2 only]* “We ask that you consent to allow your trial data to be joined up with your GenV data, *[Model 2c]* if you are in both. Then both studies can answer more questions about health and other outcomes. Under strict conditions and ethical approval, data from this trial can enter GenV’s dataset, and data from GenV can enter this trial’s dataset.”	
• *[Model 2c only]* “It is possible that [you/your child] [are/is] eligible for GenV but not enrolled in it. We encourage you to enroll in GenV (Generation Victoria). This increases the value of this trial, without adding to your time. You can join GenV by *… [trial enrolls participant; trials passes contact details to GenV; parent contacts GenV directly].”*	
• *[Model 2c only – choose relevant wording]* “You can be in this trial and not in GenV, but we will be missing some information about you/your child *or* You can only take part in this trial if you are also in GenV.”	

All trials require ethically-approved consent models and wording. As noted in Fig. 3, it is plausible that trials could be undertaken via waiver, opt-out or opt-in consent models, and that opt-in/opt-out consent could be undertaken in full (all arms, ideally before randomization) or TWiCs models. For trials wholly within GenV (Fig. 3, Model 1), GenV already includes consent for data sharing with approved users. For trials alongside GenV (Fig. 3, Model 2), trials will likely benefit most if data can flow from trial to GenV and not merely from GenV to trial (for which consent is already in place) as outlined in Section 7 (Data) below. Therefore, GenV recommends that trials include wording along the lines of Table 1 b to support maximal data utility and value.

#### Randomization

Design of randomization is determined by each trial as is most appropriate to the intervention, questions and sampling, and as per best sample selection, randomization and blinding of follow-up practice at the time. Random allocation can take place before (in the case of TWiCS) or after informed/waived consent, and by GenV or by the trial, as indicated in Fig. 3. Randomization procedures will be reported in each trial’s CONSORT statement.

### Measures

#### GenV’s measures structure

**Fig. 4:**
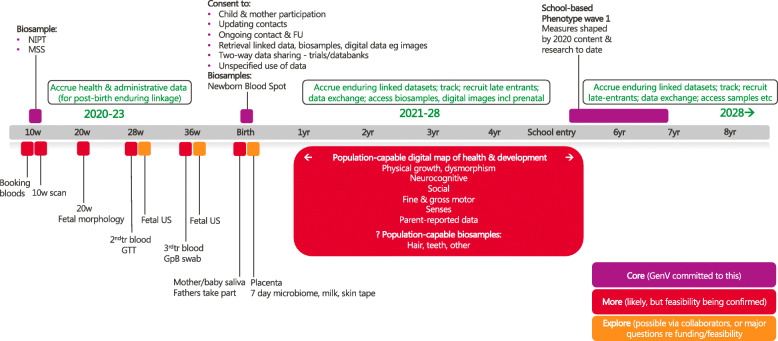
Schematic diagram showing life course accrual of parent and child data

GenV may inform trials in a variety of ways. It may provide primary or secondary outcomes, measures on which to select or stratify participants, and moderator and mediator variables. Figure 4 illustrates the range and timing of measures being explored by GenV at the time of writing, spanning linked, biosample-derived and GenV-collected data. It is expected that ultimately many, but not all, will prove feasible for GenV to include via data linkage, data collection or biosamples.

#### GenV-collected measures

Whereas birth cohorts have traditionally been purely observational in design, and focused on the discovery of longitudinal associations, GenV’s focus is on solutions to improve health and reduce the burden of disease. Testing such solutions may occur not only via trials but also natural experiments, simulation and causal modeling. All require robust outcome measures with sufficient sensitivity to demonstrate meaningful effects and an ability to quantify potential health gain when putative causal factors are targeted. The commonality of outcomes would enable comparisons of benefits and costs of different interventions for different target groups within a single dataset. A further benefit to trials is that GenV intends to collect such measures over many years, enabling trials to access longer-term data than might be possible for a single trial.

**Fig. 5:**
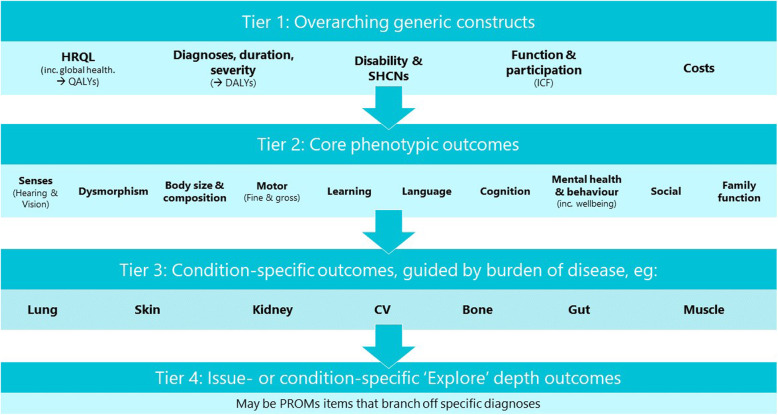
GenV’s outcomes hierarchy

GenV is planned without knowledge of what future trials may be proposed. However, GenV has developed a framework (J Wang, YJ Hu, S Clifford, S Goldfeld, M Wake: Selecting lifecourse frameworks to guide and communicate large new cohort studies: Generation Victoria (GenV) case study, submitted) and outcomes hierarchy (Fig. 5) to guide its measures selection and prioritization (Additional file 3). This framework considers GenV’s whole-of-state remit and principles of Inclusivity, Sustainability and Systematized Processes, which require low measurement burden with high ease of administration. Therefore, GenV will not collect prioritized measures that are already reliably collected and accessible via linkage sources, unless required for the day-to-day running of GenV. At the highest level, GenV will repeatedly capture overarching health and wellbeing with generic measures that have international as well as local salience: health-related quality of life (quality-adjusted life years, QALYs [31]), disease/disability burden (disability-adjusted life years, DALYs [32, 33]), requiring information on conditions, illnesses and problems that parents and children experience (International Classification of Diseases 11th Revision, ICD-11 [34]), and functional status (International Classification of Functioning, Disability and Health, ICF [35]). When coupled with service-related data, for example, regarding encounters, costs and medications, these measures would also support economic analyses.

Although comprehensive, these highest-level measures do not capture the individual traits/phenotypes that are critical to many interventions. Therefore, to support the greatest number of trials while retaining parsimony, GenV proposes to prioritize collecting outcomes included across multiple Core Outcome Sets (COS) [36] and thus already demonstrated to be of broad importance to patients, families, clinicians and policymakers as well as researchers. These span physical phenotypes (e.g., growth, body composition, dysmorphology, motor skills and senses), and mental, social, cognitive, learning and positive health. Given that GenV is targeting over 100,000 children and their parents with data from hundreds of participants daily, the only feasible way of collecting such phenotypes is remotely and digitally.

To enable capture of multiple outcomes and phenotypes, therefore, GenV is exploring developing an ‘ePhenome’, a high-throughput digital platform will let GenV measure and evaluate diverse outcomes cheaply and at very large scale, while maintaining GenV’s principles of value (including to participants) and inclusivity. We envisage that each ePhenome measurement encounter would select from a suite of ultra-short, universal-capable digital survey items, measures, images and videos. These will either be pushed universally from GenV for participants to complete remotely on any device or (for measures that meet the GenV Enhancement principle) could be collected by services within existing universal contacts.

Future funds permitting, we envisage that face-to-face school-based assessment will capture measures that require physical equipment, technical skill in administration and/or wearable devices. Such assessments may be shaped by the needs of individual trials ongoing in the years before each wave.

#### Measures collected via linkage

As well as its direct digital platform, GenV proposes to draw data wherever possible from administrative datasets. This information includes, for example, data from health, education and other providers; electronic medical records (EMR); geographic datasets; and from trials and registries. All available data will be integrated with the GenV data systems, using direct deposition, enduring data linkage and/or ephemeral Safehaven linkage processes. GenV’s ‘Victorian Child’s Lifecourse Journey in Data’ [37] lists many of the datasets that Victorian children and parents currently accrue, many of which GenV may link to in the future. GenV’s website [38] will publicly record all datasets accessed each calendar year.

#### Biosamples

Biological samples at any stage are frequently out of reach of trials due to burden or cost, especially samples that predate trial commencement. GenV is working towards the consented storage and research use of existing and new universal biosamples to the highest possible standards of conservation (including transfer to GenV’s − 80 °C autostore). Figure 4 outlines the range of samples currently being explored. It is hoped that the biosamples will span multiple tissues (e.g., blood, saliva, stool, breast milk), all participants, and multiple time points including all trimesters of pregnancy, the neonatal period and school entry. There may be potential for collaborating trials to help shape future whole-of-GenV biosample collection. Due to the depletable nature and likely small available volume of biosamples, it is highly unlikely that GenV will approve individual assays for trials, but rather will make available comprehensive biological data of the broadest value possible such as metabolomics or polygenic risk data.

#### Trial-collected data

GenV will encourage trials to collect a generic minimum dataset relevant specifically to trials, following precedents set by initiatives such as the Dutch Older Persons and Informal Caregivers Survey Minimum Dataset (TOPICS-MDS) to which over 50 projects have now prospectively contributed [39]. This small minimum dataset is to be developed collaboratively in 2020–21. Potential benefits include the ability to compare trials on common outcome metrics, evaluate effects of stacked interventions for those in more than one trial simultaneously or over time, and pooling of data for individual participant meta-analyses. Many trials will also require outcomes specific to their research questions. Once trials and GenV are agreed, the trial investigators will most likely collect samples themselves outside of GenV in dedicated visits.

### Data and sharing considerations

#### Timing of GenV data availability and implications

The data GenV hold may prove very valuable to trials because they would otherwise be unavailable due to population coverage, timeframe, jurisdiction, logistic, funding or other constraints. However, constraints could likewise apply to GenV. GenV commits to making data available on completion of a given ‘sweep’ or ‘wave,’ i.e., once all participants have provided a particular set of data. Like other major cohorts, it will generally handle data processing for its vast numbers of participants *en masse*, with benefits including efficiency and cost reduction, uniform access to technology advances (e.g., new automated scoring or assays), consistency (e.g., avoidance of batch effects/drift and of conflicting or overturned results) and completeness (unfinished data waves that do not meet the principle of Inclusivity, whereby all data are available for all participants). Each wave of GenV data collection will likely take 2 years from first to last participant to collect measures that are predicated on age milestones; thus, trials data would be available much sooner for trial interventions conducted later than earlier in a data collection wave. While some data items (e.g., straightforward PROMs (participant-reported outcome measures)) need no or minimal processing, others (e.g., image extraction) take additional time even when processing begins before the wave is complete.

Therefore, it will usually be important that trials work closely with GenV during the design and ethics approval phases to consider the timing of likely outcomes data and its implications. For example, desired GenV data may not be available sufficiently close to real-time to contribute to Data Safety & Monitoring Committees or to adaptive trial designs whereby the intervention is modulated according to therapeutic effect. Note, however, that for trials within GenV, process monitoring data (e.g., consent rates, interaction durations, data response rates) will be available promptly to optimize the trial’s compliance to its protocol.

#### Two-way data sharing

**Fig. 6:**
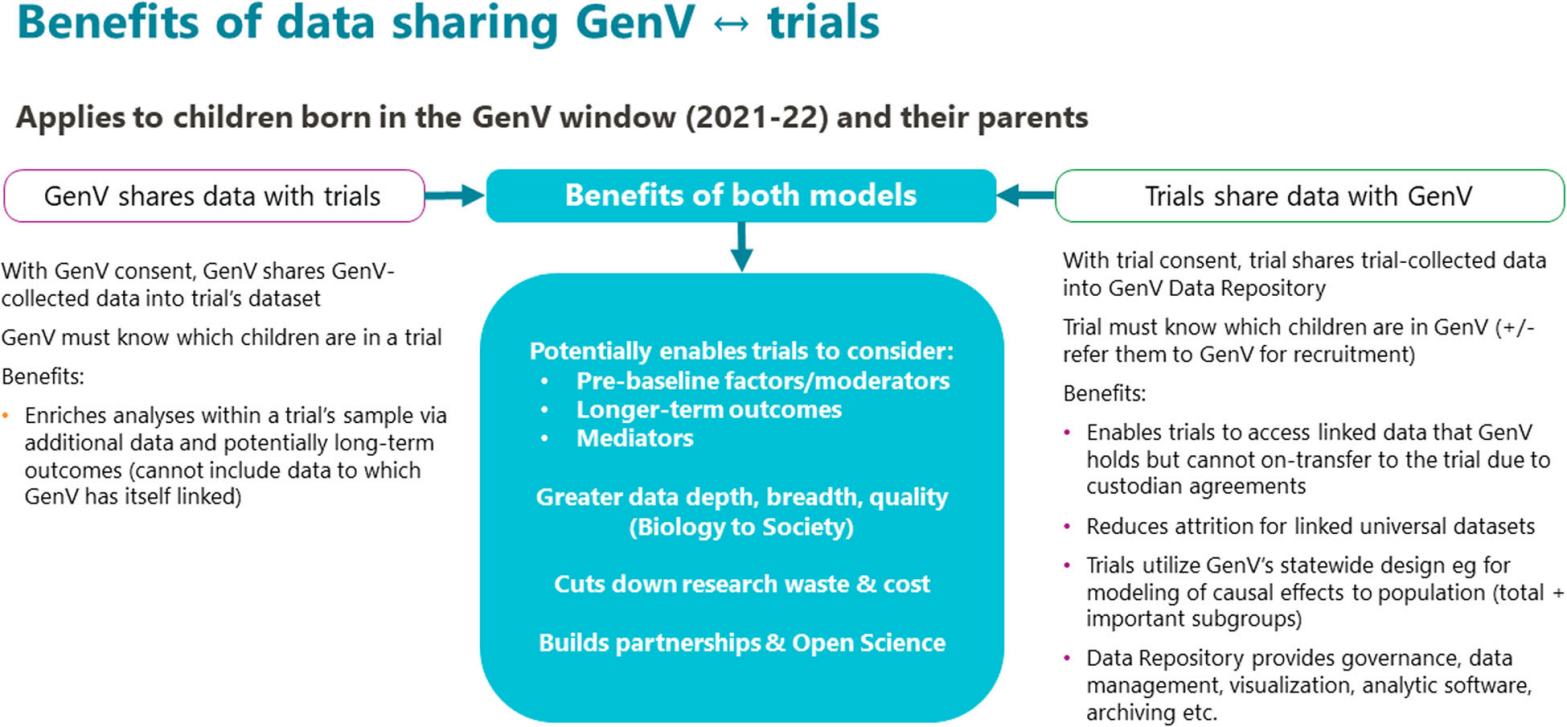
Benefits of trials sharing data with GenV and of GenV sharing data with trials

For trials conducted wholly within GenV, all data will be within its Data Repository. For trials conducted alongside GenV, data will need to be shared between the trial and GenV. Figure 6 illustrates the benefits of two-way data sharing. By GenV transferring data to trials, trials can access additional outcomes over more extended time frames, and potentially examine variation in response by moderators (such as pre-existing prospectively-collected biological or psychosocial traits) or mediators. By trials transferring their data into the GenV repository, they can access data that GenV cannot on-transfer (e.g., linked administrative datasets according to custodian agreements), model causal effects to the whole population using actual whole-population data, and combine and compare costs and benefits/utility across trials. All of these should enhance trial prominence, impact, and translation of significant findings.

Data standardization, quality control, safety and privacy applied throughout the GenV data repository will also apply to trial data that enter the GenV dataset. GenV’s data, legal, linkage and cohort personnel will provide technical support and guidance for these issues.

#### Open science

GenV is designed to be accessed by a wide variety of analysts, including researchers, service providers and policy-makers while maintaining confidentiality. Its data are intended to be an equal access resource, via the FAIR [40] and Five Safes (safe people, projects, settings, data, outputs) [41] principles, to facilitate uptake and translation. From the point at which a complete useable dataset is available to them, we propose that trialists would have exclusive access to trial data placed within GenV for 6 months (in line with non-trial studies such as the UK Biobank and the Longitudinal Study of Australian Children), with intervention/control status masked for a full 12 months.

### Sample size, analysis and reporting

Each trial will undertake its own sample size calculations, statistical analysis, and reporting according to its design and best international standards at that time. For example, it is assumed that each trial will involve a biostatistician experienced in trials, and that the trial will be analyzed and reported according to standards such as CONSORT (including the forthcoming CONSORT Extension for Trials Using Cohorts and Routinely Collected Health Data), SPIRIT and Template for Intervention Description and Replication (TIDieR) [18, 42, 43]. Trials may also access advice and support from GenV’s biostatisticians, subject to GenV funding.

### Governance and consumer/stakeholder considerations

GenV’s trials capabilities will work within the Solutions Hub, the arm of GenV that is concerned with epidemiology, science, knowledge translation and researcher engagement. The authors of this Statement of Intent comprise the current expert GenV Trials Working Group, whose Working Together Agreement is shown in Additional file 2. During 2020–21 this Group will support the governance and planning work needed to move from this Statement of Intent to a position where GenV is fully enabled to support trials as envisaged.

Although impacted by the COVID-19 pandemic, consumer and stakeholder engagement has commenced and will continue. In late 2019, GenV conducted an open web-based Focus Area survey whose analysis is nearing completion. GenV has engaged and will continue to engage widely with research and service bodies spanning health (universal, primary, secondary and tertiary), education and other sectors, who are represented on many of its Working Groups. Consumer consultation will proceed through engagement led by GenV’s Solutions Hub, including how people of Aboriginal and Torres Strait Island descent may choose to be involved.

**Fig. 7:**
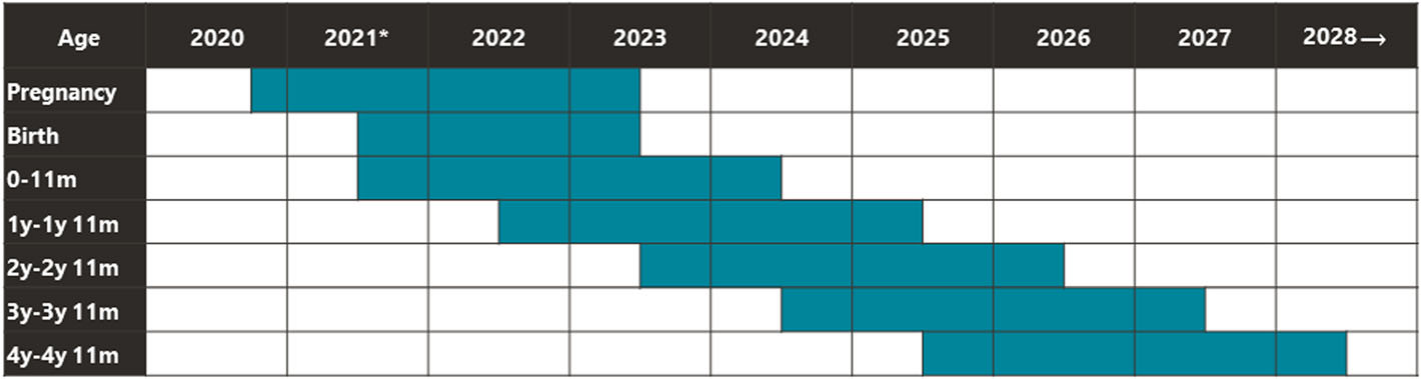
Age of children by calendar year to 2027 to assist with planning trials

We propose a formal consultation process on an annual basis, with formats yet to be determined, and focusing mainly on the major issues and opportunities for the cohort’s age and stage 2–3 years hence (see Fig. 7). This review allows enough time to plan and fund trials, put partnerships in place, and complete preparatory work such as rapid or systematic reviews supporting the need for the trial. GenV does not propose to formally limit the number of trials that a participant could enter, but rather take into consideration the needs of individual trials and apply a ‘reasonableness’ approach. If more trials are proposed or funded, than the GenV sample can accommodate, then a collaborative prioritization process will be needed to determine which can be supported.

## Discussion

### Principal findings

To our knowledge, GenV is the first mega-cohort internationally aiming to maximize its experimental as well as observational evidence via an integrated and purposive program of trials. This Statement of Intent lays out broad principles and processes ahead of GenV’s planned commencement in 2021, streamlining the integration of trials within or alongside GenV from its earliest days. Commencement of GenV immediately after COVID-19 will enable a unique and powerful platform for ongoing surveillance and response; its population reach, digital infrastructure and ability to remotely support decentralized trials place it uniquely to evaluate experimental strategies to manage the pandemic’s health, economic and social aftermath on children, families and communities during potentially lengthy partial quarantine periods and recovery.

### Strengths & Limitations

The major strength is the design of GenV itself. As a whole-population study aiming to recruit all babies born and their parents over 2 full years in the sizable and stable state of Victoria, it reaches into every metropolitan, regional, rural and remote community and every level of advantage. Its data linkage and ePhenome capacity lower the burden for both trialists and participants. The existing GenV data systems can support the activities outlined in this Statement of Intent without architectural changes. This trial-ready scaffold may empower communities that have typically lacked the necessary infrastructure to lead or join trials, especially relating to health services research and behavioral interventions. Multiple trials could be embedded, evaluating multiple interventions and identifying multiple participant groups all with a true population denominator. GenV’s Outcomes Hierarchy and time horizons should, for the first time, enable comparison on the same metrics of the lasting benefits and costs of multiple, widely-differing approaches to improve health and wellbeing. This Statement of Intent should expedite trial planning, documentation and implementation.

Regarding limitations, this Statement of Intent does not take into account the unknown success of GenV’s recruitment. This is potentially an issue if groups that could most benefit from a boost in trials-based evidence are under-represented (e.g. disadvantage, ability, minority) while noting that trials alongside GenV offer a route to redress this via later recruitment into GenV of those who initially declined or were missed. We also do not yet know whether or how much the inclusion of potential future trials in our Parent Information Statement at the time of consent will impact on GenV’s uptake rates. While smaller studies (such as ORIGINS and Born in Bradford Better Start, see below) have been generous in sharing their learnings and thus shaping our plans, we have not identified existing very large-scale studies that could highlight possible unintended consequences. We hope in due course that GenV can provide such empirical learning.

Despite the collaborative thinking underlying this Statement, detailed capabilities remain to be designed and constructed (such as processes to identify and to randomize eligible participants), some of which may only be solved once trials are in planning or underway. Some of these are discussed in Practical or Operational Issues, below. A further limitation is that GenV is not at this time funded to support trials or their administration. Such support (over and above the funds required by the trial itself) may be vital to help collaborators navigate the requirements for starting and conducting trials, especially in regional or rural hospitals and communities that do not have a robust research infrastructure. Obtaining such internal GenV ‘support’ funding will be an ongoing focus. For those who may wonder if GenV might stifle other research, we affirm that GenV has no capacity or desire to impose collaboration with clinical or other trials involving children born in the GenV birth window. We do hope for mutual awareness and communication.

### Interpretation in light of other studies

Others are recognizing the promise of longitudinal intervention cohorts to ‘stop describing and start fixing’ children’s problems [44]. The BiBBS (Born in Bradford Better Start) Experimental Birth Cohort [7] aims to recruit 5000 pregnant mothers by 2024 in inner-city Bradford, North England, and to test over 20 interventions for children’s social and emotional development using a range of designs including Trials within Cohorts (TwiCs) and quasi-experimental designs. ORIGINS [12] aims to recruit 10,000 mothers in the Joondalup region (a community in northern Perth, Western Australia) between 2018 and 2023. Within this, active participants are invited to participate in Sub-projects if they meet the eligibility criteria, with participation in some projects restricted if the outcomes overlap; at time of writing, twelve nested randomized trials are currently under way (personal communication, J Davis). Experience from both indicates a healthy appetite from researchers, policymakers and trial funders, but also that regular transparent two-way communication is vital, as are burden minimisation of and realism about timelines for trials-related data management and release unless the cohort itself is adequately funded to handle this. The HeLTI (Healthy Life Trajectories Initiative) Consortium has attracted large-scale funding from national funding bodies in its member countries Canada, India, South Africa and China in collaboration with the World Health Organization, and is well along the path of establishment [45].

Over time, GenV participants may encounter more than one trial, and therefore “stacked” interventions across childhood that respond to the issues they are experiencing. This approach is less planned than HeLTI but may mimic how children and adults accrue services naturalistically. At least one observational study has demonstrated a cumulative beneficial effect of participation in more services across childhood [46] and another that stacking multiple intervention components is cost-effective [47]. While examples are accruing of trials integrated with cohort studies under different names, such as cohort embedded RCT [48], cohort multiple RCT [49], cohort nested RCT [50] and trials within cohorts (TwiCs) [7], GenV’s freedom of design - spanning trials both within and alongside GenV - appears unusual.

### Practical or operational issues not covered in other sections

Here, we mention some of the many details that remain to be decided. Many will require resourcing both in GenV and the trials themselves. GenV will need vigilance in limiting red tape while at the same time being in a position to help prioritize, plan, standardize (e.g. measures, processes), execute and monitor trials in ways that help trials while upholding GenV principles.

We have yet to develop GenV’s internal administrative structures to achieve this (ahead of recruitment commencing in 2021). We are currently developing a framework to enable rapid, followed by increasingly deep conversations and filtering with potential collaborating trials to enable mutual understanding of likely success and benefit that does not waste time. We are also developing our mechanisms to prioritize consumer engagement and involvement, to integrate GenV-generated evidence into Living Evidence Reviews, and to progress a brief minimum trials dataset.

Despite its recognized value, data linkage remains challenging in almost all jurisdictions in Australia and worldwide, and goalposts will no doubt continue to shift. Trial ethical approval and participant consent for data sharing, sometimes years in the future, will be critical. GenV is conducting a Privacy Impact Assessment and Data Security Audit, even while knowing that practice and legislation in both continue to evolve. Victoria’s health and educational systems vary by individual practice, hospital and school, and across private/public and regional/district lines, so GenV’s statewide remit may bring challenges in terms of developing standardized interventions. Most trials require piloting; we are unsure as to whether pilots would be conducted within or outside the GenV environment and the impact of any pilots themselves.

We are uncertain as to the extent to which a trial’s research team may share GenV’s infrastructure (IT systems, data management practices) to perform trials alongside GenV, which could have practical benefits to both but would require resourcing. There may be external constraints on exactly how GenV can provide data back to trials, and the extent to which trials need to use the GenV data analysis and visualisation environments because of constraints of data custodians.

At this time, there seems to be no evidence for an upper limit of trials for a single cohort or a single participant, but their possibly interacting effects may be challenging to tease out. We do not at this time propose any limit other than participant willingness to consent, a ‘reasonableness’ lens and commitment to the GenV principles. While the inclusion of low-burden universal outcome measures makes participation in multiple trials possible both from the human and costs perspectives, GenV will need a way to monitor and prevent participant fatigue and not to overburden/compromise either GenV or the participating trials. There may be occasions where participation in one trial precludes participation in another, which will need to be considered on a case-by-case basis.

## Conclusions

This Statement of Intent outlines how the large GenV cohort can serve as a platform to increase the number, speed, range and duration of trials for parents and children. Much remains to be worked out. However, its innovative design could guide best practice for these groups which currently lack robust generalizable evidence, and whose good health and wellbeing are so vital to the functioning of populations going forward.

## Supplementary information

**Additional file 1.** SPIRIT Checklist (Microsoft Word Document (.docx)). Completed SPIRIT guideline to indicate on what page trialists would find SPIRIT-related information to consider in their trial design. However, the completed form does not apply to any actual trial or group of trials.

**Additional file 2.** Example Working Together Agreement (Microsoft Word Document (.docx)). An example Working Together Agreement (WTA) developed for the GenV Trials Working Group following GenV’s rapid evidence review of large research-led partnerships. The WTA focuses on good communication, transparency and agreement. We have included to indicate considerations for GenV and trials in developing their own WTA.

**Additional file 3.** GenV’s approach to measures selection (PNG File (.png)). GenV’s framework and outcomes hierarchy to guide its measures selection and prioritization.

## Data Availability

No data are yet available. It is intended in the future that GenV data analyses will be supported for all researchers meeting governance requirements such as the Five Safes principles. A range of materials are available at GenV’s figshare project [https://mcri.figshare.com/projects/Generation_Victoria/35822].

## Abbreviations

BiBBS: Born in Bradford Better Start
CONSORT: Consolidated Standards of Reporting Trials
GenV: Generation Victoria
HeLTI: Healthy Life Trajectories Initiative
PROMs: Participant Reported Outcome Measures
PROSPERO: International prospective register of systematic reviews
RCT: Randomized controlled trial
SPIRIT: Standard Protocol Items: Recommendations for Interventional Trials
TIDieR: Template for Intervention Description and Replication
TWiCs: Trials Within Cohorts
