# Supplementary material for: Integrating trials into a whole-population cohort of children and parents: statement of intent (trials) for the Generation Victoria (GenV) cohort
Source: BMC Med Res Methodol. 2020 Sep 24;20:238. doi: 10.1186/s12874-020-01111-x (PMC7512047; doi:10.1186/s12874-020-01111-x)
Supplement: Supplementary file 2 — Additional file 2. Example Working Together Agreement (Microsoft Word Document (.docx)). An example Working Together Agreement (WTA) developed for the GenV Trials Working Group following GenV’s rapid evidence review of large research-led partnerships. The WTA focuses on good communication, transparency and agreement. We have included to indicate considerations for GenV and trials in developing their own WTA. [file 12874_2020_1111_MOESM2_ESM.docx]

***Additional file 2: Example Working Together Agreement***

**GenV Working Together Agreement 2019: Trials Working Group**

**What is GenV?**

GenV is the largest children’s cohort ever to be planned in Australia. It is targeting all 170,000 Victorian babies born in 2021-2. By bringing together administrative, biological, clinical and GenV-collected data into a single resource GenV is building a complete picture of the health and wellbeing of Victoria’s children. GenV aims to speed up solutions for children and mid-life adults (their parents). It hopes to lead to new discoveries, new ways to predict and treat problems, and better or safer care for children with both common (like obesity, allergies and poor mental health) and less common (like epilepsy and cerebral palsy) problems. GenV converts information to action to optimize children’s wellbeing. Please see <https://www.genv.org.au/> for additional information about GenV.

We aim to build a communal resource with and for researchers, practitioners, policy and service delivery people now and into the future. We commit to equal access to the GenV resource, via the FAIR and Five Safes principles.

**Overview**

Preparatory Cohort 2020s milestones are to achieve Ethics approval (June 2019), conduct pilot work and hospitals engagement (Dec 2019), implement recruitment in a single hospital (April 2020), and roll out Vanguard recruitment in a further 3 hospitals (Sept 2020). Full state-wide recruitment is planned to run from January 2021-Dec 2022; thus, all/almost all enabling features for each Method Core must be built into GenV before January 2020.

Our seven Method Cores will help us enable different research methodologies to benefit and benefit from GenV: **Health Services**, **Trials**, **Registries**, **Population Health**, **Geospatial**, **Place and Community** and **Bioresource**.

Each Method Core Group is asked to assist GenV in developing a resource that optimally supports and ‘future-proofs’ this field of research. All activities should be filtered via the GenV Principles appended below. The priority is what must be achieved ‘now for now’ (essential to GenV’s immediate functioning) or ‘now for later’ – (if not enabled now, would be difficult or impossible to achieve later or would result in substantial missing data).

The corollary is that each Method Core will note activities/sources that are important but can be deferred until after recruitment without loss of data, utility or value (‘later for later’). An example is deferring linkage to well-curated existing data sources that are not overwritten (eg MBS).


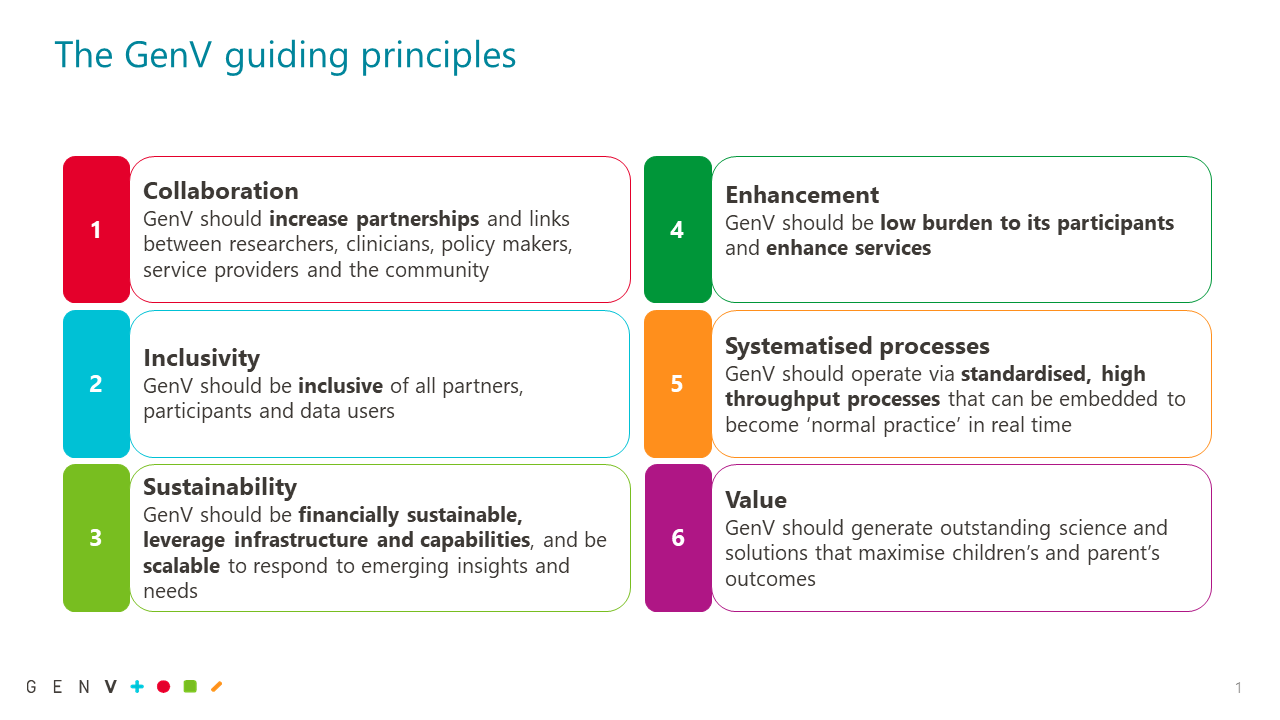
**GenV Guiding Principles**

A starting point for the Trials Group action plan from May 2019-Dec 2020 is summarized below, to be refined by the Group as it commences. This draws on needs or expectations (1) evident when developing GenV’s Protocol and Parent Information and Consent Form^1^, (2) suggested in the 2018 Methods Brainstorming Sessions^1^ and (3) thrown up in discussion with diverse stakeholders over the 2018-19. Not everything on these action plans may prove to be feasible.

We anticipate that each Group will meet at least twice over the course of 2 months. This may fully meet the immediate needs of this Method Core, or the Group may continue with meeting regularity and frequency dictated by need. Commitments by GenV and the Group are outlined below.

A parallel document is being developed for each Focus Area/Topic action plan. These will likely have a greater focus on measurement of exposures and outcomes.

**Trials Group**

**Group members**

- Co-leads – **Melissa Wake; Andrew Davidson**
- Members – **Margie Danchin; Michael Fahey; Maurizio Pacilli; Kirsten Perrett; Francesca Orsini**
- GenV – **Jessika Hu; Hayley Warren**

**Trials Group action plan/goals**

***To advise and support GenV to:***

- Develop and collect universal-capable phenotypic outcomes relevant to trials
- Develop a GenV Trials Master Protocol, if deemed appropriate
- Obtain consent to share GenV parent and child data with external trials
- Assist Ethics Committees and researchers to obtain parent and child consent to share data from external trials with GenV repository
- Consider key potential confounders common to many trials
- Summarize trial designs that may be relevant to GenV (eg cohort multiple RCTs, large simple trials, stepped wedge, master protocols) to study multiple interventions/ problems
- Consider technical issues that may arise and their solutions
- Consider ethical and governance issues relevant to trials as they pertain to GenV
- Develop working relationships with MCTC, ACTA, NHMRC Trials Centre, Pharma, children’s hospitals and other bodies active in paediatric and midlife trials
- Contribute to developing working relationships with key registries relevant to GenV’s parents and children [led by Registries Methods Group]
- Consider whether trials may be conducted within GenV, and if so mechanisms to select/stratify/randomize etc
  - If so, consider potential for waiver-of-consent and opt-out consent
- Ensure mechanisms for recruitment from trials into GenV at any age (if not at birth)

***To assist GenV to develop a Trials Statement of Intent reporting on the above***

**GenV Working Group Expectations**

**Collaboration Principles**

GenV is committed to fostering partnerships that have:

- A shared vision and goals
- Transparent and acceptable expectations
- Clear & regular communication
- Respectful relationships
- Systematized processes that optimize productivity
- Flexibility to meet the needs of each partnership

**GenV will support this collaboration by providing**

- A co-convener
- A dedicated co-ordinator
- Resources such as standardized processes, centralized communication centre and software platforms
- Access to the GenV student program and resources for supporting funding applications

**This group will support this collaboration by**

- Providing insights drawn from their expertise and experience in ***Trials*** to
- Advise on and help prioritize the *design* of GenV to maximize its utility and impact
- Help shape *research priorities* relevant to GenV 2020 children’s age and stage
- Contribute to *solving complex problems* and *improving health & wellbeing*
- Identify the needs and preferences of *GenV users* across Victoria
- Using and contributing to GenV’s data
- Promoting GenV amongst colleagues and across networks to support the health, development and welling of Victoria’s children

**Together we will**

- Draw members from a breadth of sectors: academia, policy and practice
- Be willing to share ideas to improve GenV as a resource for all
- Maintain the confidentiality of other members’ current and proposed work or research projects that may be discussed

**Specific responsibilities**

**GenV Co-lead**

- Help build momentum, enthusiasm and optimism for what could be achieved in this area
- Be a critical thinker in this area
- Act as a ‘go-to’ person for GenV in this area
- Guide and contribute to content development and review
- Introduce GenV to other experts and networks
- Be named as the Lead on relevant GenV emails and the GenV websites

**Early Career Researcher**

- Contribute to content development
- Partake in supervision of student projects arising from the group
- Actively participate and contribute to the group’s activities

**References**

1. Generation Victoria Figshare Project. [https://mcri.figshare.com/projects/Generation_Victoria/35822](https://mcri.figshare.com/projects/Generation_Victoria/35822%20) (accessed 20 March 2020).
