# Supplementary figures and images for: Integrating trials into a whole-population cohort of children and parents: statement of intent (trials) for the Generation Victoria (GenV) cohort
Source: BMC Med Res Methodol. 2020 Sep 24;20:238. doi: 10.1186/s12874-020-01111-x (PMC7512047; doi:10.1186/s12874-020-01111-x)

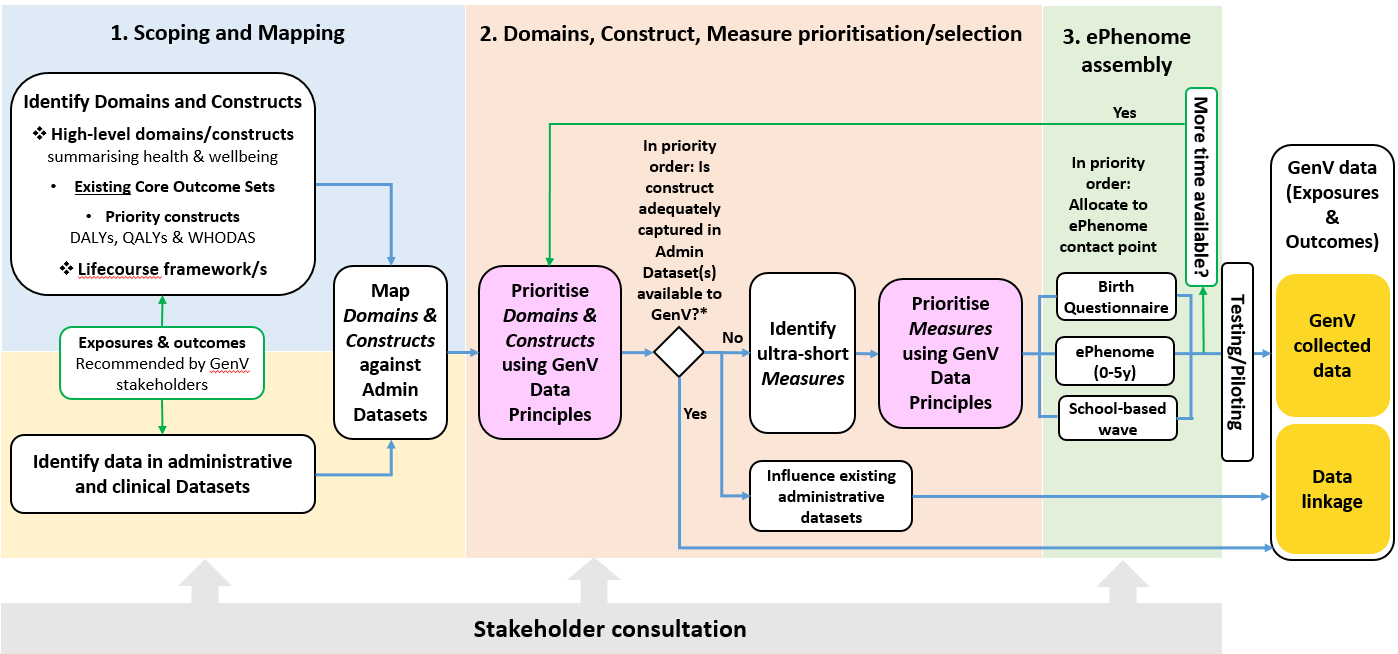

Supplement: Supplementary file 3 — Additional file 3. GenV’s approach to measures selection (PNG File (.png)). GenV’s framework and outcomes hierarchy to guide its measures selection and prioritization. [file 12874_2020_1111_MOESM3_ESM.png]
